# Theorizing Community for Sport Management Research and Practice

**DOI:** 10.3389/fspor.2021.774366

**Published:** 2021-11-19

**Authors:** Kyle A. Rich, Ramón Spaaij, Laura Misener

**Affiliations:** ^1^Department of Recreation and Leisure Studies, Brock University, St. Catharines, ON, Canada; ^2^Institute for Health and Sport, Victoria University, Melbourne, VIC, Australia; ^3^Department of Sociology, University of Amsterdam, Amsterdam, Netherlands; ^4^School of Kinesiology, Western University, London, ON, Canada

**Keywords:** community, sport management, communitarianism, social justice, sport policy

## Abstract

Community is a context for much research in sport, sport management, and sport policy, yet relatively few authors explicitly articulate the theoretical frameworks with which they interrogate the concept. In this paper, we draw from communitarian theory and politics in order to contribute to a robust discussion and conceptualization of community in and for sport management research and practice. We provide a synthesis of current sport management and related research in order to highlight contemporary theoretical and methodological approaches to studying community. We distinguish between community as a context, as an outcome, as a site for struggle or resistance, as well as a form of regulation or social control. We then advance a critical communitarian agenda and consider the practical implications and considerations for research and practice. This paper synthesizes current research and establishes a foundation upon which sport management scholars and practitioners might critically reflect on community and deliberatively articulate its implications in both future research and practice.

## Introduction

The notion of community, real or imagined, is somewhat ubiquitous within the field of sport and sport management. Whether it is explicitly addressed or more abstractly implied, understanding communities is fundamental to understanding how sport, recreation, and physical activity^1^ are engaged, promoted, managed, sold, and consumed. For example, scholars study community sport clubs, community stakeholders, community development, brand communities, social and health outcomes for community members, and even a more abstract sense of community within various sporting milieus. Despite its rather prolific use in both research and practice, the various concepts and language employed within sport management are often not explicitly articulated or deliberated. In this paper, we interrogate the concept of community in and for sport management in order to provide a synthesis of theoretical approaches and a robust conceptual discussion through which to inform both research and practice.

Our work proceeds with two sections. First, we use meta-ethnography to examine the ways that community has been engaged within the sport management literature. Drawing from various examples of research in sport management and other sport, recreation, and leisure fields, we distinguish between community as a context, community as an outcome, community as a site for struggle or resistance, and community as a form of regulation or social control. Then, drawing upon critical communitarian literature, we articulate a critical theoretical approach to studying community. While many scholars are already considering complex issues related to power, oppression, and community, we suggest that this approach will enable students, scholars, and practitioners to reframe their discussions of community by explicitly acknowledging these different ideas and engaging in critical discussions of both individual rights as well as pluralistic obligations. Our intention is for this contribution to provide a platform upon which scholars can potentially interrogate and reflect more effectively upon issues related to social justice in and through the management of sport. Drawing from this critical communitarian approach, we interrogate the meaning of community alongside the power relations inherent in management and decision-making practices, as well as the methodological processes involved in examining the intersections of community and sport management. Through this work, we aim to explore the processes and limits of community within sport management research and practice. Overall, our aim is to both consolidate discussions of community that are currently taking place in sport management, as well as to try and stimulate further discussion about innovative theoretical and methodological approaches.

## Theorizing Community

In order to frame our discussion, we must first consider the historical and philosophical underpinnings of what we now know as *community*. The study and theorizing of community (along with that of the individual and society) has long and interwoven traditions in many fields, including philosophy, sociology, anthropology, history, and political science. Much of this theorizing has involved two different yet equally important conceptions: that of the immediate community, roughly associated with the Greek understandings of the *polis* which was specific and encompassed all of the public life of individuals; and that of a universal community to which we all belong, often associated with early Roman and Christian understandings of citizenship and the church (Delanty, 2010). Contemporary work on community is often traced to the work of Tönnies (1963 [1887]), who distinguished between *Gemeinschaft* and *Gesellschaft* as two types of associative life. While these terms are not easily or directly translated, *Gemeinschaft* can be associated with horizontal relationships, regular contact, thick forms of trust, and solidarity; whereas, *Gesellschaft* is characterized by thin forms of trust, looser relationships, and vertical or hierarchical associations which are more conducive to inclusion and integration in large-scale and/or diverse societies (Arai and Pedlar, 2003; Ingham and McDonald, 2003; Delanty, 2010; Glover and Sharpe, 2020). The former foregrounds community as a territorial entity, the latter as a relational and symbolic construct (Gusfield, 1975; Cohen, 1985). In the latter, community is relatively fluid and open to change, and people may continuously weigh their options to join or leave a particular community (Delanty, 2010). In its more extreme, consumer-oriented version, community can come to reflect a (fleeting) neo-tribe (Maffesoli, 1996) or community lite (Duyvendak and Hurenkamp, 2004) released from the rigidity of the forms of organization with which we are familiar; instead, it constitutes “a certain ambience, a state of mind, and is preferably to be expressed through lifestyles that favor appearance and form” (Maffesoli, 1996, p. 98). These late (or post) modern conceptions of community echo Bauman's (2000, 2001) influential assertion that community must be understood in the context of the shift from a solid modern society to a liquid modern sociality. A core idea in Bauman's work is that we are caught up in the tension between security and freedom: the more our lives have become separated from community, the more we long to experience it.

While there is much that might be explored and unpacked with regard to historical conceptualizations of community, we turn our focus toward contemporary trends in communitarian thought and practice. We do so with a specific focus on the structures and practices that influence meaning and experiences within communities in order to explore the implications of these ideas in the context of sport management. Communitarian ideas and concepts, such as Putnam (2000) notion of social capital, have been widely discussed in the sport literature (e.g., Misener and Mason, 2006; Nicholson and Hoye, 2008; Misener and Doherty, 2012). Others have built on these discussions in the context of diverse theoretical frameworks such as social anchor theory (Clopton and Finch, 2011; Seifried and Clopton, 2013) or community capacity (Jones et al., 2018), yet few have endeavored to provide a more robust discussion of the implications of communitarianism more broadly. Some notable exceptions exist within sport and leisure studies, where scholars such as Arai and Pedlar (2003), Jarvie (2003), and Blackshaw and Long (2005) have explicitly drawn on communitarian approaches to discuss the implications of individualism and mutuality in the context of leisure theory and practice.

### Communitarian Theory and Politics

Understandings of communities in the twentieth century were undeniably complicated by the concomitant rise of globalization, technology, and (neo)liberal policy agendas. In this context, communitarianism can be understood as a “phenomenon which reveals common ground in the relationship between academic political and social theory—often of a very abstract and philosophical kind—and practical politics” (Frazer, 2000, p. 179). Communitarians are concerned with the community or the collective, rather than the individual as the unit of analysis. Defining what exactly is meant by a community, and the challenges that arise from poorly articulating these definitions, is regularly discussed within some research contexts such as community-based research methodologies (Israel et al., 2003). Frazer (2000) suggested that a strength of communitarianism is the possibility of a coalition of diverse groups (of thought and action) around the idea of a greater good. Within the context of sport, this is an important consideration given that provision and delivery of participation opportunities or engagement more broadly (e.g., as fans) often involves contributions from and collaborations between many stakeholders from the public, private, and voluntary sectors (Ferkins et al., 2010).

While there is no concise agreement on a definition or conceptualization of community within communitarian politics, Sandel (1982) distinguished between two moral and political streams of thought: those who value community rhetorically and those who value community instrumentally. That is, some value community in and of itself (e.g., for a sense of belonging), while others value community as a means for achieving other outcomes (e.g., leveraging social capital for financial or political gain). As the tensions between these two streams exist in both theory and political practice, diverse understandings of community persist ranging from simple units of identity related to place, activity, identity, or feeling (Glover and Sharpe, 2020), to much more complex and multidimensional conceptualizations. For example, Etzioni (2004) distinguished between ideas of community and identity highlighting the role of community in supporting human development and identity formation. He compared the two with the metaphor of learning how to walk (community) and learning in which direction you will walk (identity) to explicate the complexity of community as involving many identities and subcultures. Within this discussion, he offered the following:

The definition of community here followed has two characteristics: first, a web of affect-laden relationships among a group of individuals, relationships that often crisscross and reinforce one another (as opposed to one-on-one or chain-like individual relationships); and second, a measure of commitment to a set of shared values, norms, and meanings, and a shared history and identity—in short, to a particular culture (Etzioni, 2004, p. 20).

This definition is useful as it highlights both the particularistic and universal aspects of community in order to broaden the understanding of the term and appreciate its complexity. Additionally, acknowledging the social and cultural context of community allows for a more nuanced consideration of how social activities—such as sport—might be implicated in community. Given that sport occupies diverse roles and practices within different cultures (Dyck, 2000)—and by extension communities, this acknowledgment is important in order to move toward critical readings of sport and the possible social outcomes of the way it is managed and organized.

Although communitarianism is a relatively small school of political theory and practice, its scope is broad, encompassing several streams of thought, practice, and action. While discussions vary in their scope and approach, Frazer (2000) noted that communitarians hold at their core a critique of liberal schools of thought and politics which privilege individual autonomy and free market systems. Where liberal approaches value individual rights and freedoms, communitarians argue for a balance between individual autonomy and collective or pluralistic obligations (Etzioni, 2004, 2014). In short, communitarians engage with discussions of a common good to which citizens are also accountable beyond that of individual rights. This thinking is evident in the shift whereby groups and organizations recognize a list of rights *and responsibilities* in order to appreciate both conditions of reciprocity and mutuality.

Much sport management research is framed from positivist traditions and is reflective of an epistemology developed within a context dominated by (neo)liberal market principles (Shaw and Hoeber, 2017). As such, communitarian perspectives have not been widely employed despite the fresh insights they may provide, particularly in the context of community sport and recreation. While many scholars read and consider these ideas, it is difficult to reconcile and articulate a critique of free market principles in a field that is dominated by a rhetoric of neoliberalism. As noted above, we must acknowledge the potential shortcomings of a myopic view of community, particularly when its theorization is underpinned by individualism (and/or liberal perspectives) which fail to consider the implications of collectivity, mutuality, and the possibility of pluralistic obligations. Further, in order for community to function as a polymorphic concept within sport management (i.e., one with various definitions and applications which scholars can agree or disagree with), it requires scholars to articulate the philosophical and theoretical underpinnings of the conceptualizations we invoke. This position aligns with broader calls for more critical engagement with the philosophy of science and politics that underpin sport management research and practice (see Newman, 2014; James, 2018). We build on these discussions in order to explore the theoretical implications of communitarianism for sport management. To do so, we review how researchers typically invoke community before proceeding to articulate how a communitarian perspective can lend insights into sport management research and practice.

## Methods

Within sport management, community has been theorized (both explicitly and implicitly) in various ways. Diverse methodological approaches to studying individuals, groups, and societies complicate the ways that community is articulated and interrogated within the sport management literature. In preparing this paper, we sought to embrace this diversity whilst attempting to also consolidate these approaches and unpack some of their implications. We present various approaches heuristically in order to provide a synthesis of the ways in which community is presented within the sport management literature.

For this review, we drew upon a meta-ethnographic approach. Meta-ethnography is a dynamic methodology which can be used to interogate bodies of qualitative research (Doyle, 2003). The process generally involves comparing and analyzing elements of qualitative work in order to develop new interpretations (Noblit and Hare, 1988). Our meta-ethnographic approach focused on interpreting, critically synthesizing and translating conceptualizations of community from studies in sport management and related fields. This method differs from synthesis methodologies that seek to summarize or amalgamate existing findings, as our approach ultimately focuses on examining studies to re-conceptualize the way community is discussed (Doyle, 2003). Lee et al. (2015) noted that the steps to meta-ethnographic analysis can take many forms. The analytical steps to the process typically follow the steps outlined by Noblit and Hare (1988): reading, relating, translating, synthesizing, and expressing. Building on this line-of-argument approach to synthesis (Noblit and Hare, 1988), we sought to draw from the existing work to construct our overarching conceptual analysis of community. In doing so, we acknowledge that community may be theorized or interrogated in more than one way, and thus may align with more than one of the approaches outlined. We also recognize that some of the literature discussed below made reference to community, but did not seek to explicitly interrogate community. However, we contend that this does not constitute an *a priori* exclusion as irrelevant to our understandings of community.

The process proceeded as follows. The first stage, *reading*, involved identifying and organizing relevant examples of sport management research into thematic trends (e.g., community sport, brand communities, sport policy, fandom etc.). The focus of this process was reflective whereby all three authors brought forth examples, assessed, articulated, and reflected on sport management and related (e.g., leisure, sociology) literatures, and then revisited and explicitly analyzed relevant examples. This stage was iterative and involved identifying examples, discussing these trends, and then returning to the literature (particularly sport management journals such as the *Journal of Sport Management, Sport Management Review, European Sport Management Quarterly, Managing Sport and Leisure*, as well as the *International Journal of Sport Policy and Politics)* to identify and collate additional examples. *Relating* involved finding the common and recurring ways in which community was articulated and theorized in the examples put forth by all three authors. These relational patterns, or categories, were identified inductively rather than *a priori*. *Translating* involved connecting the different ways in which community has been articulated and employed (e.g., as a context, outcome, etc.) and then creating a matrix in which the relevance of examples could be organized (see Table 1 below). Finally, we *synthesized* the translations by building out our line-of-argument and *expressing* it in the paragraphs that follow. This type of review is distinguished from other research syntheses as it is it not meant to be exhaustive in scope (i.e., to review every instance where community is invoked in the literature), but rather to illustrate the application of each of the approaches and to provide tangible examples of how community has been engaged both explicitly and implicitly.

**Table 1:** Approaches to community in sport management.

**Approach**	**Description**	**Examples**
Community as context	Use in reference to a locality or geographic space or to participation in sport at the grassroots level	Research examining issues in community sport clubs or the consideration of community as a “stakeholder” for sport organizations
Community as outcome	Use in reference to something that is sought to be generated or developed through sport or sport organizations	Research examining identities, sense of community, communitas, social cohesion, social capital, networks/relationships, fandom, brand community, or social inclusion of marginalized groups in the context of sport organizations
Community as struggle or resistance	Use in reference to mobilization or collective action of groups against power structures	Research examining athlete protests or the expression of gender, sexuality, racial, or ethnic identities through sport participation or in sport organizations
Community as control	Use in reference to the ways certain (more powerful) groups maintain dominance or power over others	Research examining governmentality, active citizenship, or the role of sport or sport organizations in maintaining social order

## Findings: Approaches to Community in Sport Management

Through the meta-ethnographic process described above, we inductively developed our review of theoretical and methodological approaches around four conceptual categories: (a) community as a context; (b) community as an outcome; (c) community as a site for struggle or resistance, and; (d) community as a form of regulation or social control. As the first two categories (community as context and outcome) may appear somewhat intuitive, we attempt to delineate how they have been taken up and discussed in various sub-disciplines within sport management. The second two categories (community as a site of resistance or form of social control) are less clearly articulated. As such, we draw from a variety of literatures and examples to situate and explain these categories. We review each of these categories in turn before advancing a discussion of a critical communitarian approach. Importantly, we acknowledge that these approaches are not mutually exclusive in their application, and that scholars and practitioners may draw from various conceptualizations in their work and practice. In the sections that follow, we tease out the distinctions in order to inform a robust discussion of the philosophy and theories that underpin this work.

### Community as Context

The first conceptualization of community evident within sport management literature is that of context. By this, we refer to the idea of space, geography, or the neighborhoods in which people reside. Community in this sense, is used to describe grassroots participation opportunities including both structured and unstructured sport participation. It is also used to describe how the outcomes of sport organizations are experienced by a range of actors (e.g., development through sport or corporate social responsibility initiatives). Therefore, community as a context, is either about a physical space for participation (e.g., a local sport club, facility, or municipality) or a level of participation (e.g., in regional or nationally organized opportunities). The community context is relevant for discussions of sport development where researchers and policy makers have grappled with the longstanding tensions between goals of elite development and mass participation (Sam, 2009; Stenling and Fahlén, 2009). Further, authors have acknowledged the messiness of sport in community contexts, particularly with regard to questions of how to effectively account for and manage diverse forms of sport participation which are (and are not) engaged by diverse members of a community or society (Jeanes et al., 2018).

There are various ways in which community is invoked as a context within sport management research. Authors have explored the way that urban and rural environments shape the nature of sport involvement and the potential outcomes of participation (Svensson et al., 2017; Clutterbuck and Doherty, 2019; Rich and Misener, 2019). Less explicitly, *community sport* is also used as a broad category to qualify the context of research. For example, a 2013 special issue in the *Journal of Sport Management* focused on community sport and much of the work therein drew heavily on this conceptualization of community as a context. As described in the introduction to the special issue, community sport is understood as “the grassroots foundation of a country's sport system, and where most people engage in organized sport” (Doherty and Cousens, 2013, p. 419). The papers in that special issue addressed topics such as social inclusion (Frost et al., 2013; Maxwell et al., 2013), as well as the creation of organizational culture (Mills and Hoeber, 2013), processes of organizational resilience (Wicker et al., 2013) and (non)change (Stenling, 2013) in local sport cubs/organizations. As noted by many of the preceding authors, working in sport policy and development requires an invocation of community as an important context for sport. With the emergence of using sport as a tool for achieving a range of social policy initiatives (domestically and abroad) and the concomitant rise in a sport for all policy discourse (e.g., see Skille, 2011; Lusted, 2014) these discussions have become more complicated. As such, a contextual conceptualization of community brings about questions related to participation levels and distribution, diverse demographics, and the lived experiences of diverse groups in various sporting contexts. The conceptualization of community as context, therefore, provides an important distinction for thinking about sport (for) development, policy implementation, and access to sport participation opportunities.

### Community as Outcome

Another way in which community can be conceptualized is as an outcome that can be built, developed, or strengthened in and through sport. This approach is broad, incorporates many concepts and methodological approaches, and often draws on an understanding of community which equates it, in one way or another, with a set of identities, relationships, or feelings. Community outcomes therefore can be conceptualized as a variety of concepts including networks (Misener and Mason, 2006; Misener and Doherty, 2012), social identities (Kristiansen et al., 2015), and heritage or nostalgia (Ramshaw and Gammon, 2005). These concepts are implicated in the construction of fleeting communitas (Ingham and McDonald, 2003), a sense of community (Warner et al., 2013), social cohesion (Sabbe et al., 2020), or brand communities (Woolf et al., 2013).

One of the most prolifically discussed community outcomes is social capital. Entire volumes (e.g., Nicholson and Hoye, 2008) are dedicated to exploring the myriad of ways in which sport can be examined through a lens of social capital—from policy discourses, to the implications of infrastructure, volunteers, and experiences of marginalized groups. Within these analyses, authors have employed various conceptualizations of social capital. Most notably, Putnam (2000) concepts of bonding and bridging social capital are often invoked to qualify the nature of relationships which can be generated through participation in sport and more broadly in leisure programming. For example, Skinner et al. (2008) discussed the use of sport and leisure opportunities as a tool for disadvantaged citizens to develop networks and a sense of connectedness. This work is demonstrative of an explosion of research focusing on sport as tool for developing particular social outcomes such as social capital in marginalized communities. The work of Welty Peachey et al. (2015a,b) focused on a large sport for development event where the processes associated with liminality, communitas, and ultimately social capital development were a result of building relationships, learning, and enhanced motivation to support others. Despite these positive connotations of social capital as an asset for community, several authors have called for more nuanced approaches to studying social capital which might engage longitudinal and critical approaches to understanding the processes associated with sport and the development of relationships (Misener and Mason, 2006; Kitchin and Howe, 2013).

Within sport management, a variety of theoretical and methodological advances relate to community as an outcome. For example, several researchers have engaged in social network analysis to examine the many ways that relationships manifest, can be measured, and represented through sociograms (e.g., Hambrick et al., 2019; Barnes et al., 2021). Researchers have also conceptualized a psychosocial sense of community that can be measured and examined in relation to various participation and management activities. For example, Warner et al. (2013) discussed the development of the Sense of Community Scale, which incorporates “seven mechanisms that define sense of community within sport for participants” (p. 351). This approach draws extensively from community psychology literature (e.g., McMillan and Chavis, 1986) to propose a model which conceptualizes community as an outcome to be measured. It is considered to be influenced by mechanisms such as common interests, social spaces, leadership opportunities, and competition (Warner et al., 2012).

Sport marketing scholars have done extensive work at the intersection of fandom, identity, and community to develop the idea of brand communities. For example, Heere et al. (2011) problematized the interplay between competing identities (associated with various brand communities), and Katz et al. (2018) explored how complex relationships between fans, their teams, and each other can be understood to predict behavior. Recently, Naraine et al. (2021) examined how networks of communication form around hashtags in social media campaigns which, they suggest, are important antecedents of the development of fan identifies and brand communities that are important for relationship building and two-way engagement. These developments elucidate the complex and intersecting ways that identities, relationships, reciprocity, and communication are all implicated in development of outcomes related to community.

### Community as Struggle and Resistance

In critical sport management research, community has been framed as a site for struggle and resistance. Contributing to the expansive literature on dynamics of social inclusion and exclusion in sport, scholars have examined collective resistance to, and disruption of, power relations within sporting communities. By this, we refer to forms of community-based mobilization and collective action that aim to bring about social change in sports cultures or organizations. These efforts are typically instigated by and for community groups that have historically faced discrimination and disadvantage in sport and society, and their allies. This has historically involved (combinations of) variegated communities such as women, Indigenous peoples, Black and Minority Ethnic (BME) communities, people with disabilities, and LGBTQ2S activists. Their campaigns and everyday practices aimed at enhancing inclusivity in sport frequently adopt a form of strategic essentialism (Spivak, 1990); that is, an essentializing and to some extent a standardizing of their communities' public image, thus advancing their in-group identity in a simplified, collectivized way that downplays internal diversity and complexity (Eide, 2010).

Several authors have produced valuable insights into how sport can serve as a site of resistance. For example, Shaw and Hoeber (2003) identified the way that gendered discourses shaped knowledge about leadership roles in national sport organizations, and how these discourses might be challenged by working toward more equitable conditions for women in these organizations. There is also a notable tradition of research that examines the role of BME sports spaces as symbolic and practical sites of community mobilization and cultural identity production for specific BME communities, within the wider context of racism and exclusions in predominantly white mainstream able-bodied sports structures (Singer, 2005a; Long et al., 2009; Bradbury, 2011). Beyond the grassroots level, there is a growing body of research that explores community dissent and resistance surrounding sport mega-events. Community activism and resistance to host city bidding and staging for sport mega-events has been one such focus of research (e.g., Boykoff, 2014), while other authors have critically examined LGBTQ2S activism at the Olympic and Paralympic Games (Lenskyj, 2014; Sykes, 2016). For example, Sykes (2016) has analyzed Pride Houses at the 2010 Vancouver Winter Olympics, which showcased LGBTQ2S athletes and provided support services for LGBTQ2S athletes and spectators. Sykes discussed the complexities and contradictions of the Pride Houses as a “new form of sporting settler homonationalism” that was founded upon, and reproduced, settler colonial discourses about participation and displacement of Two-Spirit youth and Indigenous people (Sykes, 2016, p. 54). In another recent study, Quinn et al. (2020) examined the way sporting events that purport to be inclusive of athletes with a disability, serve to reinforce norms of able-bodiedness through discursive practices and spatialized approaches to sport services. In this case, where sport managers unanimously believed the model of integration was a fitting approach to be inclusive, those with direct sporting experiences (athletes and coaches) critically discussed the complexity of this practice and need to collectively resist these “unifying” narratives.

Finally, research has explored how marginalized community groups may use sport mega-events as an impetus or platform for mobilization around non-sport causes such as human rights or workers' rights. The recent study by De Lisio et al. (2018) on Rio de Janeiro's sex workers is noteworthy in this regard. These authors discussed the everyday strategies of struggle and resistance that sex workers used to navigate local authorities in search of new economic opportunities and rights in the lead-up to and during the 2014 FIFA World Cup and 2016 Olympic Games. In a similar vein, Braye et al. (2013) explored how disability activists saw the 2012 Paralympic Games as a site of resistance regarding the narratives of ability, power, and promise. These Games came on the heels of funding cuts for disability services in the UK and thus served as a site to reinforce the politics of disablement reinforcing narratives of (dis)empowered communities (Purdue and Howe, 2012). Collectively, these examples demonstrate the importance of a more critical approach to community which allows specific groups to mobilize and disrupt broader power structures in sport organizations and society more broadly. However, as noted above, examinations of struggle and resistance may serve to essentialize identities within marginalized groups or position them in competition with others for access to resources.

### Community as Regulation and Social Control

Community is not only a site for struggle and resistance, but also a form of social control and regulation. Neoliberal transformations in public policy, in particular, have reconfigured how we think about and act toward community building. The contemporary policy focus devolves much of the burden of social responsibility to individuals and civil society actors, who are expected to take responsibility for their own well-being and that of their communities (Spaaij, 2013). According to Rose (1999), the shift of social responsibility to individuals for their own communities is representative of a mode of government that may be termed *governing through community*:

[C]ommunity is not simply the territory of government, but a *means* of government: its ties, bonds, forces and affiliations are to be celebrated, encouraged, nurtured, shaped and instrumentalized in the hope of producing consequences that are desirable for all and for each (Miller and Rose, 2008, p. 93).

Governing through community represents the creation of a non-political sphere of civil society that is supposedly free to govern itself and take responsibility for its own future (Herbert-Cheshire, 2000). This tendency was evidenced in, for example, Spaaij's (2009a) study of voluntary sports clubs in rural Australia. The author suggested that sport clubs serve vital community building functions within the context of profound economic and social changes that have affected rural Australia, including privatization, economic restructuring of agriculture, and retrenchment of public policy provisions.

In sport and recreation, the notion of community as control arguably reveals itself most clearly in the myriad sports-based development programs that target poor and marginalized (and often urban) communities, which are often perceived by policymakers and funders as threats to social order (Spaaij, 2009b; Coakley, 2011). Sports-based interventions in these areas are frequently underpinned by a control-based approach to community. Examples includes Midnight Basketball programs in the United States (Hartmann, 2016) and comparable programs in other countries (Hartmann, 2015; Ekholm and Dahlstedt, 2020). While these programs seek to assist with the social development of marginalized young people, they also serve as a form of social control and regulation by serving as a means to keep “at-risk” youth off the streets and out of trouble, and to civilize. For example, in his research on a sport-based intervention targeting marginalized youth in Rotterdam, Spaaij (2009b) contended that sport “is increasingly becoming a substantial aspect of the neoliberal policy repertoire of cities like Rotterdam aimed at generating social order in disadvantaged inner-city neighborhoods” (p. 263). He found that the program trained participants in becoming normalized, regulated neoliberal subjects; that is, to assimilate them into the existing social structure, rather than seeking to change that structure. As such, these examples demonstrate that sport can be invoked as a way of maintaining order within communities by regulating actions, understandings, and ultimately acceptable forms of membership.

Another aspect of community as control that has been foregrounded in sport research is the way communities can enforce internal social control and regulation (e.g., within a sports organization). Affiliative ties and bonds of obligation with community members not only comprise vital resources, but also create impediments and constraints that limit community members' expectations, opportunities, and access. In other words, ties that bind can also keep you down. A clear example is the institutionalized practices and cultural norms in many sports clubs that constrain girls' and women's access to participation and leadership positions, and devalue or trivialize their achievements (e.g., Burton, 2015). There are numerous studies in sport that show the ways that cultural hegemonies of sport perpetuate the white, male, heteronormative, able-bodied model, and serve as a form of social control by regulating access to various realms of sport (e.g., Anderson, 2009; Jeanes et al., 2020). Several examples of hegemonic control within sport organizations were explored in a special issue of *Sex Roles* (Cunningham and Sagas, 2008; Fink, 2008). For example, Knoppers and Anthonissen (2008) examined the ways that gendered managerial discourses related to instrumentality and emotion structured the nature of work for senior managers in Dutch national sport organizations. These authors highlight the important note that taken-for-granted beliefs and practices within organizations can serve insidious roles in restricting and allowing participation of various individuals within organizations.

Collectively, our framework above highlights the different ways in which community is conceptualized and employed. Next, we turn our attention toward to a critical theoretical approach to community and how it may enhance the existing perspectives in sport management research and practice.

## Discussion: Toward a Critical Theory of Community

As noted above, community can be valued rhetorically in a way that assumes it is a universally positive construct. However, some of the literature reviewed in the preceding sections suggests that a blind acceptance of community may serve to mask the politics inherent in communities and silence the voices of diverse community members. Indeed, several traditional social structures and activities (such as the nuclear family unit, or community sport and recreation) are imbued with values of sexism/gender roles, racism, and homo/transphobia. Therefore, adopting a critical perspective of community may allow researchers to unpack the complex social dynamics involved in community life. In order to inform this approach, much can be drawn from a social justice paradigm and feminist critiques of community. While both communitarians and feminists share a critique of the extreme individualism of (neo)liberal political agendas, their grounds for doing so and proposed solutions or alternatives are quite different (Weiss, 1995). Where communitarian discussions center around balancing autonomy and collectivity or the preservation or return to traditional values of community, a more critical perspective of communitarianism questions whose autonomy is privileged, whose input is heard and considered in the collective, or which of these traditional values are acceptable in a socially just and equitable community. Rather than a traditional, idyllic, and tightly knit homogenous community, Young's (1995) suggested that community might be understood as an unoppressive city space that is constituted by “openness to unassimilated otherness” (p. 253), or a politics of difference. In short, a critical communitarian perspective is concerned with the power and politics of community and how this can be shifted or radically changed. Young (1995) politics of difference offers a theoretical tool which is helpful to problematize the complex social dynamics involved in sport, recreation, and leisure in and for community (Allison, 2000), and to deal with the deeper underlying causes of injustices within these sectors and within research on/with them (Floyd, 2014).

### Communitarianism and Sport Management

Few scholars have examined sport and recreation management explicitly through a communitarian lens, and even fewer from a critical communitarianism perspective. Notably, Jarvie (2003) applied a communitarian approach to discuss community activism and decision making around a pool and recreation facility in Glasgow, Scotland. Jarvie (2003) discussion highlighted the tensions between ideas of community and individualistic/free-market service provision in the context of a municipal sport and recreation facility in a large urban center. In a different context, Mair (2009) discussed the role of curling clubs in community life in rural Canada. Her analysis suggested that these sport clubs offer important third places, away from both home and work, which are dynamic and fluid community institutions as they offer a context for shared leisure experiences in a social context where private and individual leisure activities are becoming increasingly prevalent. These two investigations raise important questions about the possibility of managing sport in and for community in the context of neoliberal policy agendas which prioritize individuals over the collective or communal responsibilities. The work of Arai and Pedlar (2003) offered an example of challenges to the discourse of individualism and suggests that a communitarian perspective of leisure as a focal practice could allow for more nuanced analyses of outcomes for the collective rather than outcomes for individuals.

### Critically Assessing Community in Sport Management

Drawing upon the arguments outlined above, we suggest that a starting point for discussions informed by a critical communitarian approach might be how issues of social justice are inextricably bound up in the sport-community relationship. While these questions are by no means new, they can help to consolidate and advance contemporary scholarly debates. For each of the aforementioned categories, we pose questions for researchers and practitioners, and consider management implications at the intersection of community and sport management. The proposed directions are summarized in Table 2.

**Table 2:** Future research directions for community in sport management.

**Approach**	**Future research directions**
Community as context	Mapping experiences of diverse groups in diverse contexts
	Thick descriptions and contextual analyses
	Understanding the role of social, cultural, and political environments in shaping sport management practice
Community as outcome	Perceived/changing outcomes of sport and sport organizations
	Longitudinal analysis of community outcomes
	Community-based and participatory approaches to research
Community as struggle or resistance	Impacts of social movements on sport management
	How sport and sport management may be leveraged as a platform for change in and outside of sport
	Interrogating processes of knowledge production
Community as control	Critical examinations of the role of sport in community
	Changing models of governance in sport

First, we must continue to reflect on the politics of defining community as a context while simultaneously segregating sport participation based on age, gender, ability, class, and other identities. How is *community* reflected if sport, in this context, serves to divide and subdivide individuals according to so many lines of separation? In short, a community context includes many individuals who agree, disagree, look alike, look different, hold homogenous views of the world, and who fundamentally disagree on a range of issues. As scholars and practitioners, we might consider who is represented in our organizations and research, as well as who is not. Critically assessing the makeup and power structures of organizations and communities may provide an important foundation for educational and policy directives (at various levels) related to equity, diversity, and inclusion in sport. In some cases, the use of the label *community* sport may be contributing to the ambiguity of the term (see Blackshaw, 2008; Torchia, 2020) and we might consider how we can use more precise terms like *participatory* sport, *recreational* sport, *non-competitive* sport, or *grassroots* participation opportunities. While this discussion can seem like a matter of semantics, the discourses we use reflect important nuances about power, privilege, access, and opportunity in the work of sport scholars and sport managers.

As scholars have highlighted (e.g., Stenling, 2013), the organization of sport in communities requires deep and fundamental shifts if organizers and managers are to effectively serve diverse participants and diversify their offerings. Future research opportunities in this area are broad as they may consider the complex nexus of intersections related to individual and community identities and the social, cultural, and political contexts in which they develop. Here, deeply contextual analyses or thick descriptions (Geertz, 2008) will help to elucidate the role of community in shaping the way sport is organized, managed, and structured in and for different contexts. Indeed, research approaches (e.g., ethnography, institutional theory, etc.) which consider not only managerial processes, but the broader contextual factors that shape, enable, and constrain these processes are integral to mapping a robust understanding of community as context. From a critical perspective, future research may continue to interrogate how sport organizations may be implicated in existing power structures within community contexts and the impacts this has on shaping experiences of diverse community members.

Considering the prevalence of work seeking to develop outcomes related to community, a critical framework raises similar concerns about how outcomes are measured and evaluated. There are methodological and ethical issues with attempts to measure community as an outcome when these associated constructs involve various ephemeral and enduring qualities for diverse groups in society. As such, further scrutiny on how these theories are constructed and tested is required in order to more fully understand how community is experienced by diverse people. A reliance on (post)positivist approaches to research (Shaw and Hoeber, 2017) and methodologies which fail to consider temporality or the impacts of social change on individual and collective understandings of networks, relationships, or identities, will limit our understandings of communities and how they may shift, change, and be developed (Misener et al., 2021).

As both sport and community are deeply implicated in processes of social change, future research should continue to map the ways that perceived outcomes related to community are realized through sport and sport organizations. Although important recent theoretical advancements related to community have been developed through longitudinal analyses (e.g., Putnam's social capital), there is a paucity of research at the intersection of sport and community engaging longitudinal research designs. Future work using this type of analysis may yield particularly interesting insights related to sport and community for diverse population groups. To consider how diverse community members may understand and value community-related outcomes, researchers may consider the use of community-based, participatory, and action-oriented research methodologies which seek to engage community members throughout the research process (Israel et al., 2003). These methodological approaches may be particularly useful for engaging communities who may not have traditionally been centered in sport management research.

Thinking about sport as a platform for resistance may require a consideration of how social and organizational change can open up space for multiple and diverse perspectives—sometimes from the same *community*. This may require recognizing the (im)possibilities of reconceptualizing sport for equity seeking groups, and that representation is not a panacea for inequity (Shaw, 2007). That is, we must consider who gets to determine what counts as sport and sport management, what legitimate management processes and practices look like, and who is able to exercise power and authority in the processes of deciding. This difficult process may look to the work of scholars such as Stewart-Withers et al. (2017) or Chen and Mason (2019) who have advocated for decolonizing research approaches in sport management or Kobayashi et al. (2013) who explored the intersections of sport and diverse cultural understandings of community (i.e., *wantok*).

The theoretical and practical implications of this departure are vast, and beginning to be explored in the sport management literature (e.g., Singer, 2005b; Newman, 2014). Future research examining community as a site for struggle and resistance should consider how sport is implicated in social movements and how resistance is enacted both within sport organizations as well as through sport and sport organizations with a view of broader change in communities. Through this work, scholars and practitioners may engage with ontological and epistemological questions related to how knowledge about sport and sport management is constructed and the implications of these processes in late modern societies characterized by social acceleration and uncertainty (Rosa, 2013).

Finally, building on our discussion of social control and regulation, we suggest a need to critically explore the ways in which support and self-determination of marginalized citizens can be created in and through sport management. Rather than organizing sport as a way to address “problems” in communities, we need to continue to critically assess how community structures may be constructing or contributing to inequities in our social environments. This requires a recognition of the role of the norms, values, and social practices within communities and organizations (Shaw and Hoeber, 2003; Fink, 2008; Knoppers and Anthonissen, 2008; Burton, 2015). We must continue to ask how the construction of community may be experienced differently by different folks in these organizations.

In this regard, future research might critically interrogate the role of sport programming as well as research processes which seek to evaluate programs and organizational operations. The evidence provided through research is inherently political and examining the role of this research in broader policy agendas is critical in understanding the role of community as a mechanism of social control. Future research is this space may also continue to examine the intersections of public, private, and civil society organizations who are increasingly working across sectoral boundaries and ultimately changing the way the sport is understood and managed. Broader changes in governance models of sport, including the impacts of the COVID-19 pandemic, will inevitably lead to changes in the way sport is practiced and understood by community members and these changes may have important implications for sport management scholars and practitioners.

## Conclusion

In this paper, we built on contemporary literature to interrogate some of the intersections of community and sport management. We reviewed the ways in which research has articulated community as a context, an outcome, a site of struggle or resistance, and as a means of social control. Further, we introduced a critical communitarian perspective and provided commentary on how this theoretical approach might inform future research in the field and the managerial practices that research informs. This commentary advances the existing foundation upon which future scholars and practitioners might critically consider their work and the claims about the role of sport organizations and their management with regard to the various conceptualizations of communities. We encourage scholars to consider the array of methodological, theoretical, and epistemological approaches to understanding community and the implications they may bring for understanding both the theory and practice of sport management.

## Author Contributions

KR, RS, and LM all contributed to the conceptualization, analysis, and writing of this conceptual paper. All authors contributed to the article and approved the submitted version.

## Conflict of Interest

The authors declare that the research was conducted in the absence of any commercial or financial relationships that could be construed as a potential conflict of interest.

## Publisher's Note

All claims expressed in this article are solely those of the authors and do not necessarily represent those of their affiliated organizations, or those of the publisher, the editors and the reviewers. Any product that may be evaluated in this article, or claim that may be made by its manufacturer, is not guaranteed or endorsed by the publisher.

## Footnotes

^1^In this paper, we are concerned with sport, recreation, and physical activity in a broad sense. Henceforth, we use the term “sport” as a shorthand to refer to all of these.
